# Cryptosporidiosis caused by *Cryptosporidium parvum* subtype IIdA15G1 at a dairy farm in Northwestern China

**DOI:** 10.1186/s13071-014-0529-z

**Published:** 2014-11-27

**Authors:** Zhaohui Cui, Rongjun Wang, Jianying Huang, Haiyan Wang, Jinfeng Zhao, Nannan Luo, Junqiang Li, Zhenjie Zhang, Longxian Zhang

**Affiliations:** College of Animal Science and Veterinary Medicine, Henan Agricultural University, Zhengzhou, 450002 China; International Joint Research Laboratory for Zoonotic Diseases of Henan, Zhengzhou, 450002 China

**Keywords:** *Cryptosporidium parvum*, Outbreak, Dairy cattle, SSU rRNA, gp60

## Abstract

**Background:**

*Cryptosporidium* spp. are zoonotic parasites responsible for diarrhoeal diseases in animals and humans worldwide. Cattle are the most common mammalian species in which *Cryptosporidium* is detected, with pre-weaned calves considered to be reservoirs for zoonotic *C. parvum*. In October 2013, severe diarrhoea was observed in 396 pre-weaned calves at a farm in the Ningxia Autonomous Region of Northwestern China. 356 of the infected calves died despite antibiotic therapy.

**Findings:**

252 faecal samples were collected from the investigated farm. The identity of *Cryptosporidium* species was determined by polymerase chain reaction (PCR) restriction fragment length polymorphism (RFLP) analysis, and by DNA sequence analysis of the small subunit (SSU) rRNA gene. *C. parvum* was subtyped using sequence analysis of the 60 kDa glycoprotein (gp60) gene. The highest infection rate of 83.3% (40/48) was seen in 2–3-week-old calves with diarrhoea, corresponding to the age at which animals died. Three *Cryptosporidium* species were identified, including *C. parvum* (*n* = 51), *C. bovi*s (*n* = 1), and *C. ryanae* (*n* = 1). All *C. parvum* isolates were further identified as subtype IIdA15G1.

**Conclusions:**

*Cryptosporidium parvum* was likely to be most responsible for diarrhoea and death. This is the first report of a cryptosporidiosis outbreak caused by *C. parvum* IIdA15G1 in Chinese dairy cattle.

## Background

Species of *Cryptosporidium* are important zoonotic parasites that infect a wide range of vertebrate hosts, including humans [1]. There are extensive genetic variations within the *Cryptosporidium* genus. In addition to 27 recognized species of *Cryptosporidium*, more than 70 *Cryptosporidium* genotypes with no designated species names have been described [2–5]. *Cryptosporidium* can be transmitted by the faecal-oral route, *via* either direct contact or ingestion of contaminated food or water [1].

Cryptosporidiosis, caused by *Cryptosporidium* infection, is of great concern because of associated economic losses and the public health significance in humans. Diarrhoea is a typical clinical sign of human and animal cryptosporidiosis; dehydration, fever, nausea and anorexia are sometimes symptoms in infected hosts. Over 200 water-borne, food-borne, person-to-person, and zoonotic cryptosporidiosis outbreaks have been recorded [6]. The most famous cryptosporidiosis outbreak occurred in Milwaukee (Wisconsin, USA), where 403,000 people were infected through contaminated drinking water [7]. In recent years, cryptosporidiosis outbreaks have been reported in several European and American countries [8–11]. Molecular techniques have shown that *C. parvum* is the predominant species in cryptosporidiosis outbreaks, accounting for 50.8% of cases among 325 water-borne outbreaks of parasitic protozoan diseases worldwide [6].

Cattle are the most common mammalian species in which *Cryptosporidium* is detected; pre-weaned calves are considered reservoirs for zoonotic *C. parvum* [12]. Of 14 *C. parvum* subtype families (IIa, IIb, IIc, IId, IIe, IIf, IIg, IIh, IIi, IIk, IIl, IIm, IIn, and IIo), IIa and IId are the two major zoonotic subtype families in animals and humans, while IIc and IIe are anthroponotic subtype families [13]. Other subtype families of *C. parvum* are occasionally seen in humans and other animals [2,14].

In China, *Cryptosporidium* infections have been reported in domestic animals, wild animals, and humans [13–18]. A cryptosporidiosis outbreak was noticed in a paediatric hospital in Shanghai [19]. In the current study we have briefly described a cryptosporidiosis outbreak that occurred at a dairy farm in the Ningxia Autonomous Region (Northwestern China). The infection rate and species distribution of *Cryptosporidium* were also determined.

## Materials

### Cryptosporidiosis outbreak

The investigated farm, located in the Ningxia Autonomous Region of Northwestern China, was established in July 2012 with 7000 dairy cattle (2600 calves and 4400 adult cattle). The breeding cattle were introduced from Australia and Uruguay, while forage was produced in the United States. Previously, vaccination was not done against rotaviruses, coronaviruses, and *E. coli*. Before this outbreak, *Cryptosporidium* oocysts were not detected using conventional faecal examination by microscopy. In October 2013, severe diarrhoea was observed in 396 pre-weaned calves on this farm. 356 of the calves, in particular the 2–3-week-olds, died despite being treated with antibiotics. We collected 30 faecal samples from calves with diarrhoea and subjected them to testing for gastrointestinal pathogens [Rota, Corona, *E. coli* F5 (K99), Crypto] using the commercial Digestive Kit (Real Bio-technology, Qingdao, China). One month later, the symptom of diarrhoea gradually disappeared in calves.

### Ethics statement

During specimen collection, all animal experiments were conducted in accordance with the Chinese Laboratory Animal Administration Act 1988.Prior to experiment, the protocol of the current study wasreviewed and approved by the Research Ethics Committee of Henan Agricultural University (License no. 2014-018).

### Sampling and microscopy examination

To determine infection rate and species distribution of *Cryptosporidium*, we obtained 252 faecal samples: 158 were from pre-weaned calves, 55 from post-weaned calves, and 39 from heifers and adults. Fresh faecal samples for each animal were collected immediately after defecation on the ground. *Cryptosporidium* oocysts in faecal samples were concentrated using Sheather’s sugar flotation technique, with samples from pre-weaned calves subjected to formalin-ethyl acetate sedimentation [14]. *Cryptosporidium*-positive samples were determined by microscopy and stored in 2.5% potassium dichromate at 4°C prior to DNA extraction.

### Molecular identification

Genomic DNA was extracted from *Cryptosporidium*-positive faecal samples using the E.Z.N.A. Stool DNA kit (Omega Biotek Inc., Norcross, GA, USA) according to the manufacturer’s recommended procedures. *Cryptosporidium* species were determined by polymerase chain reaction (PCR) restriction fragment length polymorphism (RFLP) analysis, and by DNA sequence analysis of the small subunit (SSU) rRNA gene [20]. Subtyping of *C. parvum* was conducted using a nested PCR technique targeting the gp60 gene, which encodes the 60 kDa glycoprotein [2,21]. Amplicons were sequenced on an ABI PRISMTM 3730 XL DNA Analyzer using the Big Dye Terminator v3.1 Cycle Sequencing Kit (Applied Biosystems, Foster City, CA, USA). Sequence accuracy was confirmed by two-directional sequencing and by sequencing a fresh PCR amplicon if necessary. Sequence alignment was carried out using ClustalX 1.83 (ftp://ftp-igbmc.u-strasbg.fr/pub/ClustalX/). Representative nucleotide sequences were deposited in GenBank (Accession numbers KM215133-KM215134, and KM873712-KM873713).

## Findings and discussion

Using the Digestive Kit, we found that nine samples were positive for *Cryptosporidium* and four were positive for rotavirus. None of the samples analyzed with the Digestive Kit were positive for coronavirus or *E. coli* F5 (K99).

Microscopy examination revealed the overall prevalence of *Cryptosporidium* to be 21% (53/252). The infection rate was 31 (49/158), 7.3 (4/55), and 0% (0/39) for pre-weaned, 3–11-month-old, and >1-year-old animals, respectively. The highest infection rate of 83.3% (40/48) was seen in 2–3-week-old calves that had severe diarrhoea. Three *Cryptosporidium* species were identified based on RFLP and sequence analysis of the SSU rRNA gene; there were 51 *C. parvum* isolates in pre-weaned (*n* = 48) and 3–11-month-old (*n* = 3) calves, one *C. bovis* isolate in a 3–11-month-old calf, and one *C. ryanae* isolate in a pre-weaned calf. All *C. parvum* isolates were successfully amplified at the gp60 locus, with sequencing analysis suggesting they were the IIdA15G1 subtype.

Diarrhoea is common in calves worldwide, and is considered a major cause of productivity decline and economic losses in the cattle industry [22]. Previous studies have indicated that rotaviruses, coronaviruses, *E. coli*, and *C. parvum* are all contributing factors to diarrhoea in calves. However, *C. parvum* is believed to be the primary cause of calf diarrhoea, and is a potential zoonotic agent [23,24]. Results from the Digestive Kit in combination with high *Cryptosporidium* prevalence (83.3%) in 2–3-week-old calves with diarrhoea were strong indicators that *C. parvum* was likely most responsible for diarrhoea and death.

An overall infection rate of 21% on this farm was higher than that observed in Heilongjiang (15.0%), Henan (13.0%), and Shannxi (3.4%) provinces in China [14,25–28]. The highest level of prevalence (83.3%) in 2–3-week-old calves was similar to that seen in previous studies [29], and was consistent with the morbidity and mortality of calves in this age group.

The predominance of *C. parvum* infection in pre-weaned calves seen in this study was highly similar to those seen in most studies conducted in other countries [2,14,27,28]. It is known that *C. parvum* is a major etiological agent for sporadic cases, or water- and food-borne disease outbreaks in humans worldwide [9,30–33]. As a major reservoir of *C. parvum*, pre-weaned calves might play an important role in zoonotic infections [12].

Previously, the *C. parvum* subtype IIdA15G1 has been detected in cattle, sheep and goats in Iran, Malaysia, and Spain [34–37]. It has also been found in humans from the Netherlands, Australia, Iran, Malaysia, and India [37–41]. The IIdA15G1 subtype identified in this study was unlikely to have arrived *via* Uruguay or the United States because the *C. parvum* IId subtype family has never been found in the Americas [13]. It is also unlikely that IIdA15G1 was introduced from Australia because *C. parvum* has not been detected in Australian breeding cattle. Thus, the source of the cryptosporidiosis outbreak could be attributed to *C. parvum* contamination in the area surrounding the farm. The IIdA15G1 subtype has been found in rodents in China, with *C. parvum* isolates from animals all belonging to the IId subtype [14,18,27,28]. Generally, IId is a major zoonotic subtype family reported in Europe (Hungary, Germany, Portugal, Sweden, Ireland, Spain, Belgium, Romania, the United Kingdom, the Netherlands, Slovenia, and Serbia and Montenegro), Asia (Kuwait, Iran, Jordan, India, Malaysia, and China), Africa (Egypt and Ethiopia), and Australia [13,14,42,43].

In comparison with cryptosporidiosis outbreaks in humans, only a few outbreaks have been reported in animals, including goats in Brazil and Oman [44,45], foals in New Zealand [46], cockatiels in Japan [47], stone curlews in the United Arab Emirates [48], alpaca crias in the United States [49], chelonians in Australia [50], and rabbits in Poland [51]. The findings from our current study provide further information regarding cryptosporidiosis outbreaks among animal populations.

In conclusion, this is the first report of a cryptosporidiosis outbreak in Chinese dairy cattle. The *C. parvum* IIdA15G1 subtype was responsible for the reported outbreak as determined by genotyping and subtyping tools. Further studies are required to improve our understanding of the transmission and public health significance of *Cryptosporidium* species in China.

## Conclusions

This is the first report of a cryptosporidiosis outbreak in Chinese dairy cattle. The *C. parvum* IIdA15G1 subtype was responsible for the reported outbreak as determined by genotyping and subtyping tools.
